# Streptavidin/biotin: Tethering geometry defines unbinding mechanics

**DOI:** 10.1126/sciadv.aay5999

**Published:** 2020-03-25

**Authors:** Steffen M. Sedlak, Leonard C. Schendel, Hermann E. Gaub, Rafael C. Bernardi

**Affiliations:** 1 Lehrstuhl für Angewandte Physik and Center for NanoScience, Ludwig-Maximilians-Universität München, Amalienstr. 54, 80799 Munich, Germany.; 2 NIH Center for Macromolecular Modeling and Bioinformatics, Beckman Institute for Advanced Science and Technology, University of Illinois at Urbana-Champaign, Urbana, IL 61801, USA.

## Abstract

Macromolecules tend to respond to applied forces in many different ways. Chemistry at high shear forces can be intriguing, with relatively soft bonds becoming very stiff in specific force-loading geometries. Largely used in bionanotechnology, an important case is the streptavidin (SA)/biotin interaction. Although SA’s four subunits have the same affinity, we find that the forces required to break the SA/biotin bond depend strongly on the attachment geometry. With AFM-based single-molecule force spectroscopy (SMFS), we measured unbinding forces of biotin from different SA subunits to range from 100 to more than 400 pN. Using a wide-sampling approach, we carried out hundreds of all-atom steered molecular dynamics (SMD) simulations for the entire system, including molecular linkers. Our strategy revealed the molecular mechanism that causes a fourfold difference in mechanical stability: Certain force-loading geometries induce conformational changes in SA’s binding pocket lowering the energy barrier, which biotin has to overcome to escape the pocket.

## Teaser

Single-molecule force spectroscopy reveals how the mechanical stability of a widely used biomolecular complex varies fourfold.

## INTRODUCTION

The concept of using mechanical forces to shape materials is considered a universal knowledge. The result of applying mechanical force is recognizably dependent on the direction of the force. For instance, a transversal force can easily bend an iron bar, while a much higher longitudinal force is needed to deform it. The same holds for biological materials: A timber wood log exhibits the same behavior except that wood fibers can be torn apart if the force is strong enough. A far more intriguing question is how mechanical forces affect single biomolecules. The chemistry at high forces can be really unexpected. Recently, it has been shown that an array of hydrogen bonds can be as strong as a covalent bond when a macromolecular system is designed such that all hydrogen bonds have to be broken at the same time to separate a protein-peptide complex (*1*). The directionality of forces can regulate key biological activities. For instance, some genetic diseases cause mutations in mechanoactive proteins that, in turn, lead to notable phenotypic differences in humans (*2*). In a simulation study, Best *et al.* (*3*) investigated the unfolding of a small protein domain for different pulling directions. However, studying different force-loading geometries experimentally on the single-molecule level is not straightforward, and little is known about how larger protein complexes behave under mechanical load applied from different directions. Modern force spectroscopy investigates these issues. The streptavidin (SA)/biotin interaction is abundantly used in biotechnology, with a particular use as a molecular anchoring system in single-molecule force spectroscopy (SMFS) experiments. It is thus important to fundamentally understand its mechanics and its dependence of the force-loading geometry.

To this day, tremendous effort has been invested to probe the mechanical strength of a single SA/biotin interaction. Previous studies, varying in instrumentation and immobilization strategies, found a wide range of unbinding forces for the SA/biotin complex (*4*–*9*). The underlying molecular mechanism for the mechanical stability of this complex has also been extensively investigated using computational tools (*8*–*11*). To consolidate the discrepancies in the reported unbinding forces, we investigated the unbinding of biotin from different SA subunits with total control of subunit geometry (*12*) by building on state-of-the-art site-specific immobilization strategies (*13*), parallelized atomic force microscopy (AFM)–based SMFS of different molecular species on a single sample surface (*14*), and the development of SA mutants with defined valences (*15*).

## RESULTS

### AFM-based SMFS on the SA/biotin system using SA of different valences

**Fig. 1:**
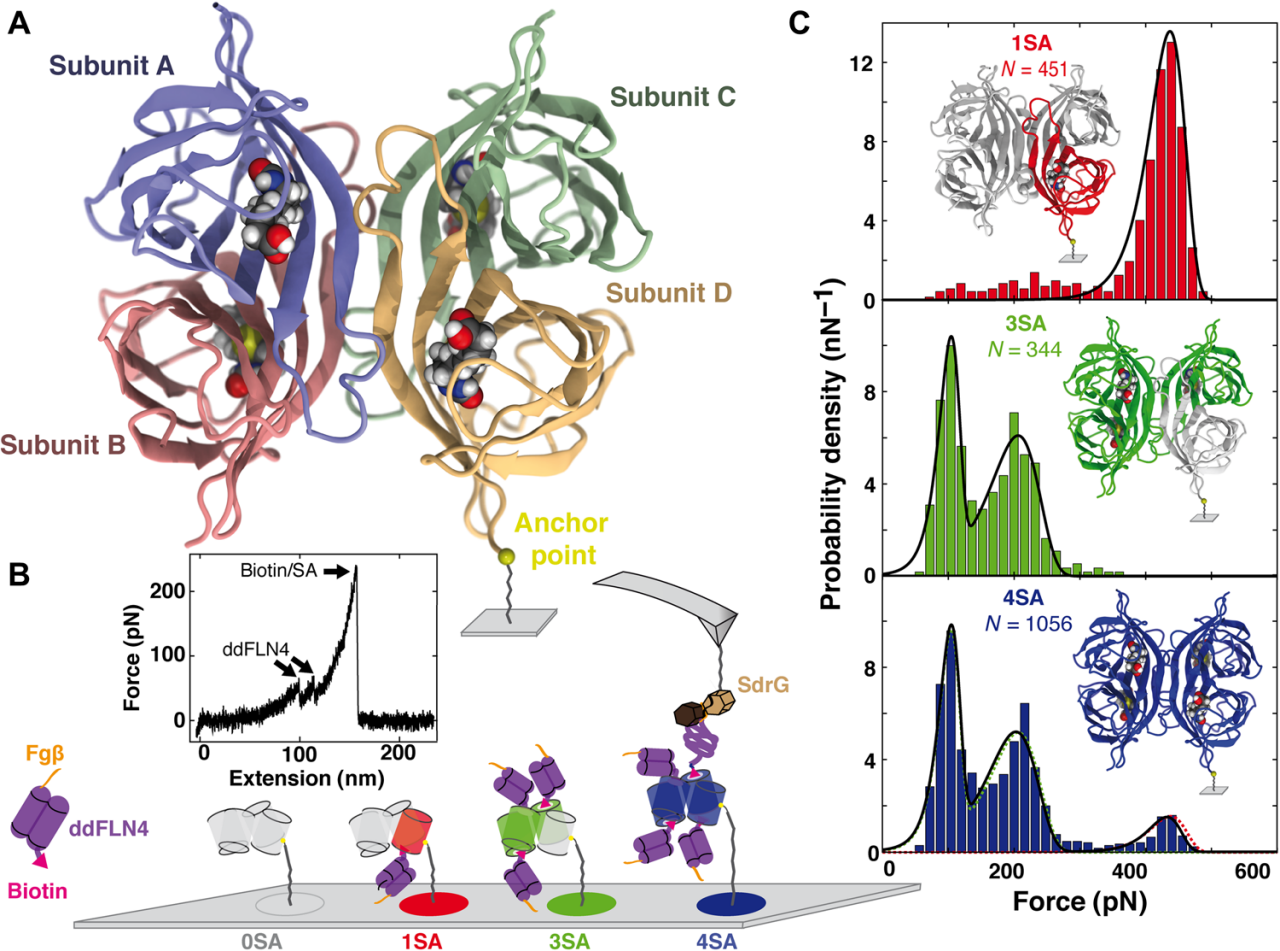
Force spectroscopy of the SA/biotin complex with different valences. (**A**) Crystal structure of SA. SA comprises four subunits, each consisting of a β barrel into which a biotin molecule can be bound. At the C terminus of subunit D, a unique cysteine is used as anchor point for site-specific covalent immobilization by maleimide–polyethylene glycol (PEG) linkers onto a functionalized glass surface. (**B**) Combining nonfunctional (light gray cylinders) and functional subunits (colored cylinders) allows preparation of SA of different valences. These different SA variants are immobilized at different areas on the surface: 0SA (gray), 1SA (red), 3SA (green), and 4SA (blue) are all examined with the same cantilever. Biotinylated ddFLN4 (purple) with an N-terminal Fgβ peptide (orange) is added to the solution. While biotin (magenta) binds to SA on the surface, the Fgβ peptide can bind to the SdrG domain (brown) immobilized on the cantilever. Retracting the cantilever, ddFLN4 unfolds, and biotin is pulled out of the binding pocket, while the force is recorded. A typical force extension trace is shown in the inset. (**C**) After sorting the force curves for specific interactions, i.e., for those showing the specific unfolding pattern of ddFLN4, unbinding force histograms are plotted and fitted with Bell-Evans distributions: 1SA (red) is fitted with a single Bell-Evans distribution. To fit 3SA (green), a double Bell-Evans distribution is needed. 4SA (blue lines) is fitted with a triple Bell-Evans. Furthermore, a combination of distributions of 1SA and 3SA can be fitted (red and green dotted lines).

To prepare SA of different valences, we expressed functional and nonfunctional (*15*) SA subunits separately and assembled them in defined ratios (fig. S1). Combining one subunit that contained a purification tag and a unique cysteine for surface attachment with three other identical subunits, we created four different SA constructs: nonfunctional (0SA), monovalent (1SA), trivalent (3SA), and tetravalent (4SA) SA. Our protocol (see Materials and Methods) does not allow for preparation of a divalent SA with distinct orientation of the functional subunits relative to the cysteine residue. In the following, the subunit that contains the unique cysteine, i.e., the one that is attached to the surface in SMFS, is always denoted as subunit D. The other subunits are denoted accordingly, as given by the crystal structure in Fig. 1A.

For AFM-based SMFS experiments, the four different SA variants were covalently and site-specifically tethered in millimeter-separated spots on the same glass slide (Fig. 1B). This allowed circumventing inconsistencies of cantilever calibration and measurement conditions because all SA variants were probed with the same cantilever that enabled reliable and precise comparison of the resulting unbinding forces (*14*). Comparing rupture measurements from different setups performed under different conditions may be delicate, since stiffness of the pulling device, the retraction velocity, and the type and length of linker molecules can affect rupture forces observed in SMFS (*16*–*18*).

As previously established (*9*), cantilever clogging was avoided by using a proxy receptor ligand system: The adhesin SD-repeat protein G (SdrG) from *Staphylococcus epidermidis* and its binding partner, a short peptide from human fibrinogen β (Fgβ), were used because their rupture forces far exceed those of SA/biotin interaction (*1*). Data with covalent attachment of biotin to the cantilever tip are provided in fig. S2. To unambiguously identify single-molecule unbinding events, we used *Dictyostelium discoideum*’s fourth filament domains (ddFLN4) (*19*, *20*), with an N-terminal Fgβ peptide and a C-terminal biotin to establish a molecular link between SdrG on the cantilever tip and SA on the sample surface (Fig. 1B).

### Different unbinding forces for different binding geometries

**Fig. 2:**
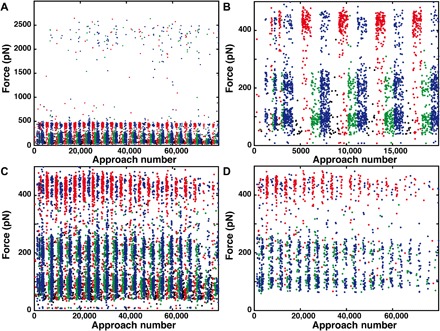
Course of a SMFS measurement. (**A**) For all interactions between cantilever tip and surface (higher than 50 pN), the rupture forces are shown. Interactions recorded on the 0SA spots are shown in black, 1SA spot in red, 3SA spot in green, and 4SA spot in blue. Most rupture forces are smaller than 500 pN. The rare events above 2000 pN correspond to the unbinding of the Fgβ from SdrG. (**B**) Zoom-in on the start of the measurement. The biotinylated Fgβ-ddFLN4-biotin construct was added after 1200 approach-retraction cycles; before that, only a few nonspecific interactions occur. At first, in every 250 curves, a different surface area is probed and then every 1000 curves. (**C**) Zoom-in on forces lower than 500 pN. The unbinding from the different SA subunits manifests itself in the clustering of unbinding events around 100, 220, and 450 pN. (**D**) Specific interactions only. For all interactions, for which the distinct two-step unfolding pattern of ddFLN4 is observed directly before the complex ruptures, the unbinding force is plotted.

To measure the unbinding forces, the same AFM cantilever tip was repeatedly probing the different SA variants immobilized at different spots on the same surface (Fig. 2). Of 80,000 binding attempts, around 10,000 retraction traces showed interactions with forces higher than 50 pN (Fig. 2A). About one-fifth showed the distinct two-step unfolding pattern of ddFLN4 (fig. S3) before the rupture of the SA/biotin complex (Fig. 2D). These data are plotted as histograms of unbinding forces in Fig. 1C. On the surface where 0SA, the nonfunctional control mutant, was immobilized, only two events (of 20,000 attempts) showing a ddFLN4-like force curve pattern were observed demonstrating the low level of nonspecific interactions in the assay. For 1SA, the unbinding force histogram exhibits a single, most probable rupture force of 440 pN, fitted well by a Bell-Evans distribution (*11*, *21*) for dissociation of a single bond in a single-step Markovian manner. In contrast, the unbinding force histogram of 3SA exhibits two peaks at lower forces with maxima at 100 and 210 pN. The high forces seen for 1SA do not occur for 3SA. The histogram can be fitted by a cumulative function of two Bell-Evans distributions. The unbinding force histogram of events recorded on the 4SA area reveals a combination of both, 1SA and 3SA, namely, three distinct unbinding force peaks. We find the occurrence of these different force peaks for the different SA variants to be consistent over various loading rates (fig. S4). The red and green dashed lines in the bottom panel of Fig. 1C are weighted 1SA and 3SA fits from before. Using a cumulative function of three Bell-Evans distributions results in a comparable fit. Fit formula and parameters are provided in Supplementary Notes and table S1.

Combining the functional subunits of 1SA and 3SA leads to 4SA. The same is true for the force histograms: The combination of the rupture force histograms of 1SA and 3SA resembles the histogram of 4SA. Thus, we interpret the data by attributing the different rupture force peaks in the histogram to unbinding of biotin from different SA subunits. Evidently, unbinding from subunit D can be attributed to the highest rupture force peak at 440 pN because 1SA only shows this single peak. The attachment of the tetramer to the surface via subunit D might explain the comparatively low relative frequency of this rupture force event in the 4SA histogram due to lower accessibility of the subunit D binding pocket. The two remaining force peaks thus stem from biotin unbinding from subunits A, B, and C.

Other possible effects such as heterogeneity in the binding cannot explain the different force peaks measured for the different SA variants because the occurrence of the different peaks in the histograms is clearly related to which subunits of SA are accessible for biotin. If they were caused by any kind of heterogeneity of the SA/biotin interaction itself, then the results for the different SA variants would be the same. Heterogeneity of the SA/biotin bond, as proposed by Rico *et al.* (*8*), might yet account for the shallow background of lower unbinding forces seen for 1SA.

Compared with other recent SMFS studies, the rupture forces for a single SA/biotin bond reported here are relatively high. For example, Senapati *et al.* (*22*) reported forces below 100 pN. In their study, surface attachment of SA was yet accomplished by one or several of its various amine groups (movie S1), resulting in a less defined attachment geometry and most probably on average weaker mechanical stability. With constant force measurements in magnetic tweezers, Löf *et al.* (*23*) have shown that the lifetimes of single SA/biotin bonds for SA attached by its various amines are spread over a wide range and, on average, are about 10 times lower than the lifetime of a single bond between biotin and 1SA anchored by the C terminus of its functional subunit. In the present study, we use the latter, mechanically stronger, attachment geometry to investigate the molecular roots of this discernible dependence of mechanical stability on force-loading geometry. As shown here by using the same chemistry, the same linkers, the same buffer conditions, the same AFM cantilever tip, and the same experimental setup for measuring four different SA variants, this difference in mechanical stability is inherent to the SA/biotin system; all other discrepancies between miscellaneous experiments (like different AFM cantilevers, different setups, and different buffer conditions) add up on top.

### Combining in silico and in vitro force spectroscopy reveals unbinding mechanism

To elucidate the underlying molecular mechanism, we performed all-atom constant velocity steered molecular dynamics (SMD) (*11*) simulations using the same force-loading geometry as for the SMFS experiment. Simulations of a fully solvated SA/biotin complex (fig. S5) were prepared following QwikMD (*24*) protocols and carried out with graphics processing unit (GPU)–accelerated NAMD (*25*). A wide-sampling approach was taken where hundreds of fully independent simulations were carried out, accounting for more than 30 μs of production SMD runs. For simplicity, we always anchored the molecular linker of biotin bound to subunit D [Protein Data Bank (PDB): 5TO2 (*26*) and biotin from PDB: 1MK5 (*27*)] and pulled on one of the four subunits by its C terminus. This reproduces the four different experimental force-loading geometries. Furthermore, the simulations include part of the linker, which connects biotin to the Fgβ-ddFLN4 construct (fig. S5). We found that omitting the linker yields significantly different results (fig. S6), presumably due to missing interactions between the linker and SA.

**Fig. 3:**
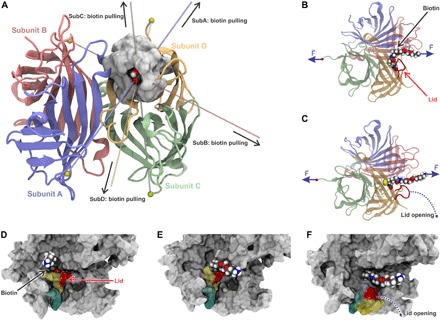
Direction dependent unbinding and lid opening. (**A**) Schematics of the force-loading geometries. To simplify MD simulations, biotin bound in subunit D (shown with surface representation) was anchored by the end of its molecular linker, while one of four subunits (A to D) was pulled by its C terminus. Colored lines indicate the four resulting force-loading directions (polymeric biotin linker is not shown). (**B** and **C**) The structure of SA stretched via its subunit C and the end of the polymeric linker of biotin bound in subunit D are shown before (B) and after (C) lid opening just before bond rupture. (**D** to **F**) Surface representation of SA shows how the stretching of biotin and its linker during subunit C pulling—from initial conformation at time 0 ns (D), to time 32 ns (E), to time 54 ns (F)—induces conformational changes in the binding pocket’s lid (colored by amino acid sequence).

During SMD simulations, because the pulling and anchoring points are gradually separated at constant pulling velocity, the complex is free to rotate into an orientation, maximizing the distance between the attachment points. This orientation defines the direction in which gradually a restoring force builds up in the molecular complex upon further separation. In Fig. 3A, the crystal structure of SA/biotin complex is depicted. For the binding pocket, a surface representation is chosen to illustrate the spatial confinement of biotin. The four colored lines connect biotin’s carboxyl group with the C termini, indicating the different initial force-loading directions. Upon stretching, the molecular linker approximately aligns along this line. SA tightly encapsulates biotin, except for biotin’s carboxyl group to which the molecular linker is covalently attached. For pulling on subunit D, which showed the strongest unbinding forces in the experiment, the initial force-loading direction points straight through the binding pocket cavity (yellow line in Fig. 3A). For the other subunits, the initial force-loading directions pierce through the binding pocket’s confinement. Upon stretching, biotin will be pushed against parts of the enclosing binding pocket. We hypothesized that this levering of biotin or the adjacent molecular linker against flexible parts of SA destabilizes the binding pocket and interferes with its structural integrity, resulting in lower unbinding forces.

### The position of the L3/4 loop is crucial for tight encapsulation of biotin

Binding of biotin to SA is mediated by hydrophobic interactions, a network of hydrogen bonds, and a conformational change in the SA subunit (*28*): A flexible peptide loop between the third and the fourth β strand (L3/4 loop) closes over the binding pocket like a lid and buries biotin inside the pocket (*29*). Calculations performed by Bansal *et al.* (*30*) showed that this conformational change accounts for about 75% of the change in free energy upon biotin binding. In the analysis of our SMD data, we therefore focused on this vital contribution of the lid to biotin binding. We propose that for the three weaker attachment geometries (anchoring of subunits A, B, or C), the L3/4 loop is, under load, forced toward its open conformation. By analyzing SMD trajectories, we observed that the lid opens up before biotin dissociation, particularly in those simulations where subunit A or C were probed. To illustrate the mechanism of force-induced lid opening, we depicted different stages of the SMD simulation (for pulling of subunit C) in Fig. 3 (B to F) and fig. S7.

### Detailed picture of system mechanics with atomistic resolution

**Fig. 4:**
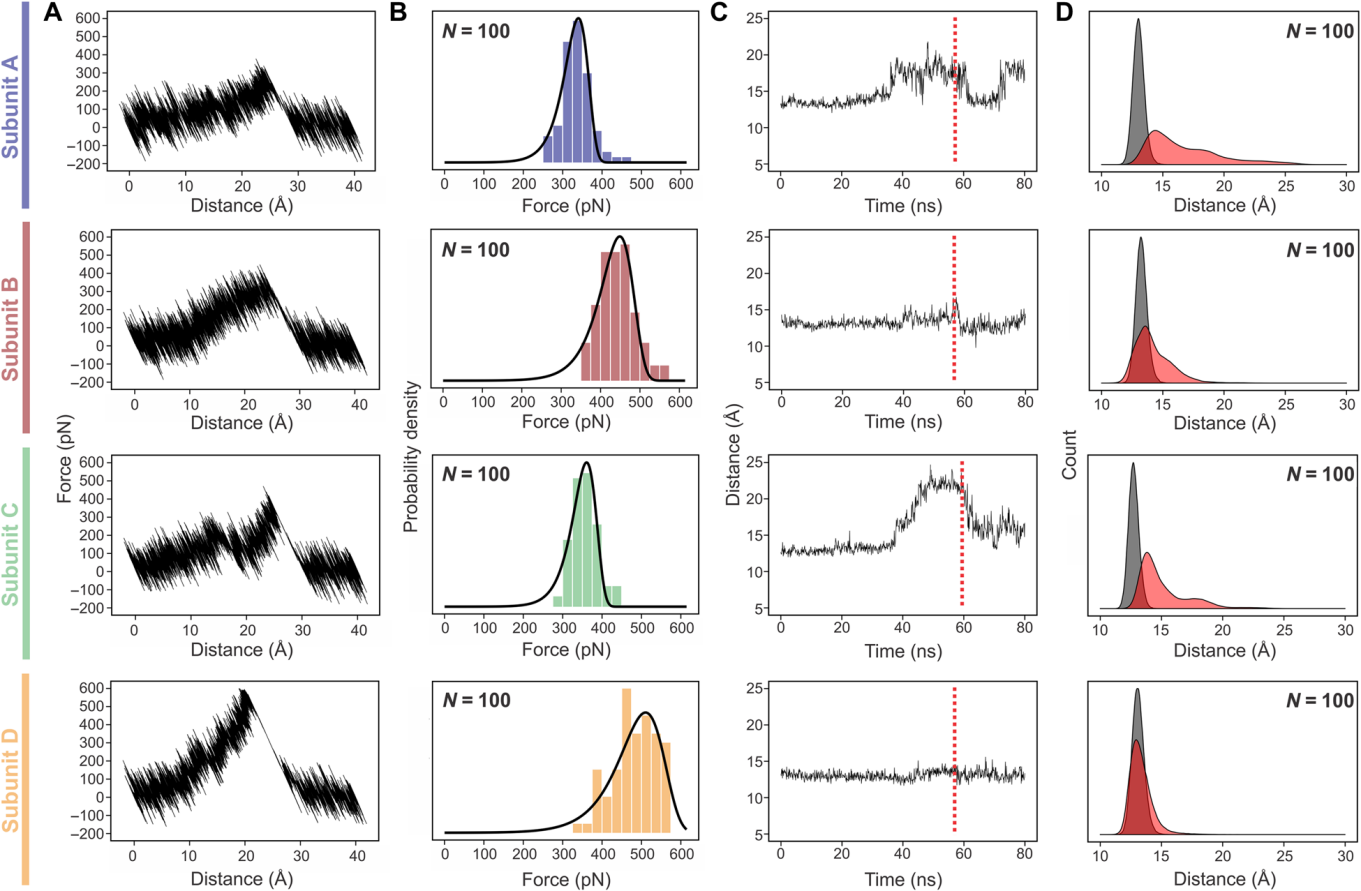
Results of SMD simulations. Pulling C termini of SA subunits while holding molecular linker of biotin bound to pocket in subunit D. (**A**) Exemplary force extension traces for the four geometries. (**B**) Resulting rupture force histograms fitted with Bell-Evans distributions. (**C**) Exemplary plots of the distance metric for the L3/4 loop opening (distance between α carbons of Gly^48^ and Leu^124^ residues) over time. The red dashed lines denote the moment at which biotin leaves the pocket. (**D**) Histograms of the distance metric for the L3/4 loop opening for the first 10 ns of the simulation (unloaded condition, gray) and for 10 ns just before the point of rupture (loaded condition, red).

Beyond this phenomenological description, the wide-sampling SMD strategy allowed statistical treatment of the SMD data (Fig. 4). Plotting rupture force histograms for the SMD data (Fig. 4B) reveals that the SMD results agree qualitatively with the experimental SMFS data: The force needed to unbind biotin from subunit D is the highest (510 pN), while the unbinding forces from subunit B are lower (450 pN). The unbinding from subunits A and C is observed at similar forces of about 340 and 360 pN. Plotting a histogram combing forces from all domains shows that subunits A and C results are nearly indistinguishable (fig. S6).

At first glance, the absolute forces measured in experiments and simulations differ quantitatively from each other. In this context, it has to be taken into account that the pulling velocity and therefore the loading rates, which in turn influence the observed rupture forces (*31*), are much higher in the simulation. To directly compare the results of simulations with those of the experiments, we plotted them as a dynamic force spectrum and fitted the data with a straight line according to the standard Bell-Evans model (fig. S4). In this framework, the difference in rupture forces between simulations and experiment are comprehensible. Solely, the rupture force for unbinding from subunit D observed in the simulations are slightly too low. Presumably, the force field parameters for biotin and its linker are less precise when compared to those for amino acids (which have been optimized over decades). Taking this and also the six orders of magnitude difference in pulling velocities into account, we can say that we also have a convincing quantitative agreement between in silico and in vitro SMFS. Both issues could be solved or at least reduced; however, requiring enormous computational resources, which as we previously demonstrated (*32*), is better spent producing more replicas and consequently better statistics.

To monitor the position of the L3/4 loop, we introduced a distance-based (Fig. 4, C and D) and an angle-based metric (fig. S8). For the former, we measured the distance between the α carbon of residue Gly^48^ (tip of the L3/4 loop) and the α carbon of residue Leu^124^ (middle of β strand β8; fig. S9). By tracking this metric over time for single representative trajectories (Fig. 4C), we found that for subunits A and C, the distance abruptly increases about 10 ns before the complex ruptures, which indicates that the L3/4 loop opens (movies S2 and S4). Subunit B exhibits a similar but much less pronounced behavior (movie S3), while for subunit D, the distance is constant up to the point of rupture (movie S5). A histogram over all 100 replicas confirms this trend (Fig. 4D): While for subunit D, the distributions at the beginning of the force loading (gray) and around the rupture (red) are almost congruent; they differ significantly for the other three subunits, particularly for subunits A and C.

**Fig. 5:**
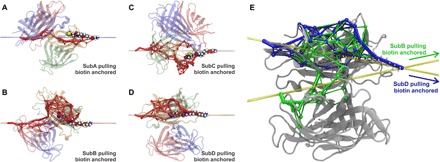
Force propagation pathways through the SA tetramer. (**A** to **D**) The force propagation pathway is shown for the different subunits close to the point of rupture. Force propagation pathways were obtained from cross-correlation–based network analysis calculated for all 100 replicas in a force-loaded condition. α carbon atoms serve as nodes that are connected by tubes of different diameters corresponding to how likely it is to have force transferred between them. SA is rotated to align the directions of force application horizontally. (**E**) Overlay of the force propagation pathway of subunits B and D. Within subunit D, the two are similar. For subunit D, a strong correlation is found between the molecular linker of biotin and the fourth β strand of subunit D, revealing a stabilization of the SA/biotin interaction pocket.

To investigate how force propagates through the receptor-ligand complex, we used a cross-correlation–based network analysis (*33*). From thermodynamic fluctuation theory, one can infer that paths with high correlation of motion can be isolated to describe the paths along which force propagates through the system (*33*, *34*). In Fig. 5, the force propagation pathways through the SA tetramer are depicted. Whereas clear differences between the four force-loading geometries are evident, one can observe that force propagation pathways for subunits B and D are quite similar within subunit D. The network model suggests that interactions between receptor and ligand are highly correlated in multiple sites of the subunit D β barrel, as it was previously shown for C-terminal pulling of subunit D (*9*). Since for force loading of subunits A, B, and C, the force has to propagate through the SA tetramer, it is in principle imaginable that not the SA/biotin interaction but the SA tetramer structure ruptures, as suggested by Kim *et al.* (*35*). While we cannot rule out such a process for our AFM-based SMFS experiments, any indication for rupturing of the SA tetramer was absent in the SMD simulations.

## DISCUSSION

Very little is known about the chemistry at the interface between biomolecules affected by mechanical forces. Here, we show that simple geometric concepts can be used to explain enormous differences in the strength of biomolecular complexes. Our findings, and the proposed mechanism of directionality dependence of force, are certainly important in a more general way and relevant for many other biomolecular complexes that encounter mechanical stress. The force resilience dependence on the tethering geometry has previously been observed by comparing N- and C-terminal tethering, revealing that some biomolecular complexes are more stable in one of these tethering geometries. For instance, bacterial adhesion in *Staphylococcus* infection has been shown to be extremely stable at the biomedically relevant tethering geometry but weak at an alternative geometry (*1*). The same was observed for cellulosomal complexes, where the ultrastable cohesin/dockerin complexes were shown to be relatively easy to break if pulled in a geometry that does not activate the complex bond (*33*). However, in all these cases, the main factor of the lower complex stability was in fact the lower stability of the protein fold in a different tethering geometry; therefore, the difference in force resilience was in fact determined by the interplay between unfolding and unbinding. Here, we show that some complexes might present completely different unbinding pathways depending on the tethering geometry while keeping the original fold. From our results and by inspecting the structure highlighted in Fig. 3A, one can quickly realize that each tethering will create different unbinding pathways. We expect that the same holds for many other biological complexes.

For the SA/biotin interaction, which has been frequently measured by SMFS, the influence of tethering geometry has so far been overlooked contributing to a wide range of rupture forces reported in the literature. In this work, we reconcile these seemingly conflicting results of previous force spectroscopy studies on the SA/biotin interaction from a more complete perspective, showing that for four different well-defined tethering geometries, the experimental unbinding forces can vary fourfold. Anchoring of SA via unspecific pulldown by reactive amines or similar groups as it is done in most commercial products might result in an even wider range of unbinding forces. Therefore, we show that the way in which SA is tethered is of critical importance for the force propagation path in the complex and thus for the mechanical stability of the SA/biotin interaction.

In summary, we show that diversity in binding forces was revealed to be caused by different force-loading geometries and that the accompanying induction of conformational changes was caused by pushing biotin against the flexible L3/4 loop of SA. We demonstrated that for SMD simulations, it is important to consider the experimental force-loading geometry and take explicitly into account molecules that may be interfering with the receptor-ligand interaction, such as the biotin linker molecule. Together, our findings encourage to reconsider how SA is tethered in future force spectroscopy experiments: With site-specific anchoring and consideration of resulting force-loading geometries, higher mechanical stability of the SA/biotin bond can be achieved in future investigations. Likewise, since biotin is attached to a molecular linker for most applications in bionanotechnology, our experimental and computational design follow the predominant scenario for assays using SA/biotin complexes and should be used to guide new developments whenever these complexes might be under mechanical stress. Therefore, by illustrating how protein mechanics of a biomolecular system depends on tethering geometry, our work not only provides a more precise protocol for single-molecule experiments but also sheds light on the fundaments of protein mechanics.

## MATERIALS AND METHODS

### Preparation of proteins

All protein sequences used are provided in Supplementary Notes. Green fluorescent protein (GFP), used for polyacrylamide electrophoresis, or ddFLN4 were cloned into plasmids for expression by T7 RNA polymerase (pET) and expressed as described by Sedlak *et al.* (*12*). Recombinant GFP and ddFLN4 proteins contain a ybbR-tag that was used for biotinylation using Sfp-Synthase as described by Erlich *et al.* (*36*).

For cloning and expression, we follow a protocol provided by Baumann *et al.* (*37*). The four different SA subunits were cloned into pET vectors. Subunits were expressed separately, denatured, mixed, and purified by Ni–immobilized metal affinity chromatography (IMAC).

For example, to obtain 3SA, we denatured nonfunctional SA subunits (with polyhistidine tag and a unique cysteine at their C terminus) and mixed them with denatured functional subunits without tags in a 1:10 ratio. After protein refolding, we used Ni-IMAC to select for SA with a single polyhistidine tag, i.e., 3SA. A complete description is given by Sedlak *et al.* (*9*).

To ascertain the number of functional subunits per SA, we added biotinylated GFP to the different SA variants and performed SDS–polyacrylamide gel electrophoresis (SDS-PAGE). The different SA variants (0SA, 1SA, 3SA, and 4SA) were mixed with biotinylated GFP. We allowed the proteins to bind to each other (about 10 min) before adding a loading buffer. Proteins were then loaded onto the Any kD Mini-PROTEAN TGX Stain-Free Protein Gel (Bio-Rad, Hercules, USA).

This protocol for preparation of SA of different valences can, in principle, be used to prepare divalent SA. Since the assembly of different subunits into the tetrameric SA is stochastic, the orientation of functional and nonfunctional subunits relative to the anchoring point cannot be controlled. Beyond that, if a unique anchoring point were desired, three different types of subunits would have to be assembled, further complicating the protocol.

### Surface preparation

Heterobifunctional polyethylene glycol (PEG) of 5000 g/mol molecular weight was dissolved to 25 mM in a 50 mM Hepes buffer at pH 7.5 and added onto an amino-silanized glass slide. During 30 min of incubation, the *N*-hydroxysuccinimide (NHS) group on one end of the PEG linker formed a stable amide bond with the amines on the glass slide. After washing off unbound PEG using ultrapure water, a silicon mask was placed on the surface and at different spots, and 10 μl of the reduced SAs dissolved in coupling buffer was added onto the surface. The SA’s unique cysteines reacted with the maleimide group on the other end of the PEG to form a stable thioether bond. A graphical illustration of the process is given in the supplementary materials of Sedlak *et al.* (*9*).

### Cantilever preparation

Bifunctional PEG of 5000 Da having an NHS group at one end and a maleimide group on the other (NHS-PEG5000-MAL, Rapp Polymere, Tübingen, Germany) was dissolved in 50 mM Hepes at pH 7.5 and immediately used to incubate amino-silanized BioLever mini (Olympus Corporation, Tokyo, Japan; spring constant from calibration after the experiment: 0.15 N/m). After 1 hour, the cantilevers were thoroughly washed in ultrapure water and then placed in 25 μl droplets of coenzyme A (CoA) dissolved in coupling buffer [50 mM NaCl, 50 mM NaHPO4, and 10 mM EDTA (pH 7.2)]. After 1 hour, the cantilevers were thoroughly washed in ultrapure water and then placed in 25-μl droplets of the Sfp reaction mix {10 μl of 10× Sfp buffer [10 mM MgCl2 and 50 mM Hepes (pH 7.5)], 5 μl of 100 μM Sfp-Synthase, 40 μl of 32.5 μM SdrG-ybbR construct (*1*), and 45 μl of MiliQ H2O}. After at least 1-hour incubation time, the cantilevers were thoroughly washed in phosphate-buffered saline (PBS) and stored in PBS. A graphical illustration of the process is given in the supplementary materials of Sedlak *et al.* (*9*).

For covalent attachment of the biotinylated ddFLN4 domain, the biotinylated ddFLN4 construct with the C-terminal cysteine was used and coupled to the maleimide instead of the CoA. After at least 1-hour incubation time, the cantilevers were thoroughly washed in PBS and stored in PBS.

### AFM-based SMFS experiments

The AFM-based SMFS measurements were performed with a custom-built AFM controlled by an MFP-3D controller (Asylum Research, Santa Barbara, USA) and a self-written routine programmed in Igor Pro 6 (WaveMetrics, Oregon, USA). The cantilevers were approached to the surface with 3000 nm/s and, after short contact (indentation of 100 pN), retracted with a constant velocity of 800 nm/s. The readout of the distance and cantilever deflection was performed at 12,000 Hz. The cantilever was retracted at 350 nm. After each approach-retraction cycle, the surface was moved 100 nm in lateral direction to expose a fresh surface area to the cantilever tip. All measurements were performed in PBS (pH 7.4) in ambient conditions. Cantilevers were calibrated following the thermal noise method as described by te Riet *et al.* (*38*).

For measurements with a second receptor-ligand system on the cantilever tip, we first performed about 1000 approach-retraction cycles to ensure the absence of unspecific interaction between the SA on the surface and the SdrG on the cantilever tip. We then placed the mounted AFM cantilever tip in PBS containing the biotinylated Fgβ-ddFLN4 construct at a concentration in the low nanomolar range for 2 min. By this, some ddFLN4 gets adsorbed to the cantilever tip. We then transferred the AFM head back onto the sample surface and continued with the approach-retraction cycles, now measuring specific interactions. An alternative approach that also worked is to directly add the diluted biotinylated Fgβ-ddFLN4 construct to the measurement buffer.

For measurements with several surface areas, where different proteins are immobilized, the cantilever tip was retracted a few micrometer from the surface after 250 to 2000 approach-retraction cycles. Then, the surface was moved a few millimeters in lateral direction so that the next surface area could be probed. The cantilever was approached automatically, and the probing of the surface continued.

### AFM-based SMFS data analysis

Using the cantilever spring constant, the optical lever sensitivity, and the *z* piezo sensitivity, the deflection voltage and the *z* piezo voltage are translated into force and distance, respectively. Then, the cantilever-bending correction is performed, and the value for zero force and zero distance are determined for each force extension trace. After denoising, each force extension trace is translated into contour length space. Detecting force peaks, force extension traces are sorted to identify those that show the correct increase in contour length corresponding to the distinct two-step unfolding of the ddFLN4 fingerprint domain. Rupture and unfolding forces for each surface area are analyzed separately and plotted as histograms.

### Molecular dynamics simulations

Using advanced run options of Visual Molecular Dynamics (VMD) (*39*) QwikMD (*24*) plugin, our in silico approach followed established protocols that were previously used to investigate mechanical properties of SA (*9*), filamins (*2*), cellulosomes (*14*), and adhesins (*1*).

### System setup

The structure of a monovalent *Streptomyces avidinii* SA (mSA) had been solved by means of x-ray crystallography at 1.65 Å resolution and was available at the PDB (PDB: 5TO2) (*26*). Because this crystallographic structure does not contain a biotin bound to the binding pocket, the structure of the tetravalent *S. avidinii* SA bound to biotin (PDB: 1MK5) (*27*), solved at 1.4 Å resolution, was used to place the biotin on to its binding site at chain D of the mSA. The PEG3 molecular linker used in the experiments was designed with VMD’s molefacture (*39*) plugin. The alignment and placing of the biotin with linker into the monovalent structure were performed using VMD (*39*) on the basis of the alignment of the aforementioned crystal structures. Using the quantum mechanics/molecular mechanics (QM/MM) tools of QwikMD (*24*), we performed a short 10-ps long hybrid QM/MM molecular dynamics (MD) simulation with NAMD (*25*, *40*) and Molecular Orbital PACkage (MOPAC) (*41*) using a 0.5-fs integration time step. The classical Chemistry at Harvard Macromolecular Mechanics 36 (CHARMM36) force field was used to represent the SA atoms, while the biotin and its linker were treated with QM at Parametric Model 7 (PM7) level (*42*). This QM/MM simulation was performed without the presence of solvent molecules and kept the SA and the biotin nonhydrogen atoms with position restraints, allowing only for the linker to search for a plausible conformation. The biotin with its linker was then parameterized for classical MD simulations using CHARMM General Force Field (*43*). Using advanced run options of QwikMD (*24*), the structure resulting from the QM/MM simulation was solvated, and the net charge of the system was neutralized in a 0.15-M sodium chloride solution. In total, about 275,000 atoms were simulated in each of the classical MD simulation. The CHARMM36 force field along with the transferable intermolecular potential with 3 points (TIP3) water model was used to describe all systems.

### Equilibrium MD simulations

All classical MD simulations were performed in GPU-accelerated XK nodes of the National Center for Supercomputing Applications/Blue Waters supercomputer using the NAMD MD package (*25*). All simulations were performed assuming periodic boundary conditions in the NpT ensemble with temperature maintained at 300 K using Langevin dynamics for temperature and pressure coupling, the latter kept at 1 bar. A distance cutoff of 11.0 Å was applied to short-range nonbonded interactions, whereas long-range electrostatic interactions were treated using the particle-mesh Ewald method. The equations of motion were integrated using the r-RESPA multiple time-step scheme (*25*) to update the Lennard-Jones interactions every step and electrostatic interactions every two steps. The time step of integration was chosen to be 2 fs for all simulations performed. Before the MD simulations, an energy minimization was performed for 5000 steps. An MD simulation with position restraints in the protein backbone atoms and biotin and linker nonhydrogen atoms was performed for 10 ns. To allow for a total relaxation of the system and to make sure biotin and its linker were stable in the SA pocket, a 100-ns simulation in equilibrium, where no external forces were applied, was performed. The MD protocol served to preequilibrate the system before the SMD simulations was performed.

### SMD simulations

With structures properly equilibrated and checked, SMD simulations (*11*) were performed using a constant velocity stretching (SMD-CV protocol) at 0.5 Å/ns. The SMD procedure is equivalent to attaching one end of a harmonic spring [with a spring constant of 1.0 kcal/(mol Å^2^), i.e., 0.69 N/m] to the end of a molecule and pulling on the end of the other molecule with another spring. The force applied to the harmonic spring is then monitored during the time of the MD simulation. The pulling point was moved with constant velocity along the *z* axis, and due to the single anchoring point and the single pulling point, the system is quickly aligned along the *z* axis. Owing to the flexibility of the experimentally used linkers connecting the domains of interest and the fingerprint domains, this approach reproduces the experimental protocol. Simulations were performed restraining the position of the terminal nitrogen of the biotin linker while pulling the α carbon of each subunit’s C-terminal amino acid residue. For all four configurations, many simulation replicas were performed in a wide-sampling approach. For each subunit pulling, 100 replicas were performed, with each of the simulations accounting for 80-ns total simulation time. In total, 32 μs of production SMD was performed.

### Simulation data analysis

Simulation force-time traces were analyzed analogously to experimental data. For each simulation, the rupture force was determined as the highest force of a trace, and the force-loading rate was determined as a linear fit to the force versus time traces immediately before rupture. Analyses of force traces and MD trajectories, except for the force propagation analyses, were carried out using python scripts taking advantage of Jupyter Notebooks (*44*). Particularly, VMD (*39*), MDAnalysis (*45*), and PyContact (*46*) were used for trajectory analysis together with in-house scripting wrappers, which collected information from all simulation replicas. Force propagation analyses were performed using dynamical network analysis, which is implemented in VMD’s Network View plugin (*47*). A network was defined as a set of nodes, all α carbons plus three atoms of the biotin and its linker, with connecting edges. Edges connect pairs of nodes if corresponding monomers are in contact, and two monomers are said to be in contact if they fulfill a proximity criterion, namely, any heavy atoms (nonhydrogen) from the two monomers are within 4.5 Å of each other for at least 75% of the frames analyzed. Filtering this network, one can investigate allosteric signaling (*40*, *47*). Allostery can be understood in terms of pathways of residues that efficiently transmit energy, here in the form of mechanical stress, between different binding sites (*33*). The dynamical networks were constructed from 10-ns windows of the total trajectories sampled every 400 ps. The probability of information transfer across an edge is set as w_ij_ = −log(|*C*_ij_|), where *C* is the correlation matrix calculated with Carma (*48*). Using the Floyd-Warshall algorithm, the suboptimal paths were then calculated. The tolerance value used for any path to be included in the suboptimal path was −log(0.5) = 0.69. As previously demonstrated by our group (*33*), Pearson correlation is ideal for force propagation calculation.

## Supplementary Material

aay5999_Movie_S2.mov

aay5999_SM.pdf

aay5999_Movie_S4.mov

aay5999_Movie_S3.mov

aay5999_Movie_S5.mov

aay5999_Movie_S1.mov

## SUPPLEMENTARY MATERIALS

Supplementary material for this article is available at http://advances.sciencemag.org/cgi/content/full/6/13/eaay5999/DC1 Fig. S1. SDS-PAGE of different SA variants. Fig. S2. SMFS measurements with direct covalent attachment of the biotinylated ddFLN4 domain to the cantilever tip. Fig. S3. Exemplary force extension traces. Fig. S4. Dynamic force spectrum. Fig. S5. Structure of biotin with the adjacent linker and illustration of the simulation box. Fig. S6. SMD force histograms. Fig. S7. Structure of the SA/biotin complex during L3/4 loop opening. Fig. S8. Angle metric for L3/4 loop opening. Fig. S9. Distance metric for L3/4 loop opening. Table S1. Fit parameters for the Bell-Evans distributions shown in the main text. Note S1. Fit parameters of Bell-Evans distributions. Note S2. Sequences of protein constructs. Movie S1. SA’s crystal structure with highlighted amine groups. Movie S2. Exemplary SMD: Holding biotin, pulling on the C terminus of SA subunit A. Movie S3. Exemplary SMD: Holding biotin, pulling on the C terminus of SA subunit B. Movie S4. Exemplary SMD: Holding biotin, pulling on the C terminus of SA subunit C. Movie S5. Exemplary SMD: Holding biotin, pulling on the C-terminus of SA subunit D.

## Appendix

View/request a protocol for this paper from *Bio-protocol*.
