# Supplementary material for: Streptavidin/biotin: Tethering geometry defines unbinding mechanics
Source: Sci Adv. 2020 Mar 25;6(13):eaay5999. doi: 10.1126/sciadv.aay5999 (PMC7096159; doi:10.1126/sciadv.aay5999)
Supplement: aay5999_SM.pdf [file aay5999_SM.pdf]

Science Advances
AAAS

advances.sciencemag.org/cgi/content/full/6/13/eaay5999/DC1

Supplementary Materials for

Streptavidin/biotin: Tethering geometry defines unbinding mechanics

Steffen M. Sedlak, Leonard C. Schendel, Hermann E. Gaub*, Rafael C. Bernardi*

*Corresponding author. Email: gaub@lmu.de (H.E.G.); rcbernardi@ks.uiuc.edu (R.C.B.)

Published 25 March 2020, *Sci. Adv.* **6**, eaay5999 (2020)
DOI: 10.1126/sciadv.aay5999

The PDF file includes:

Fig. S1. SDS-PAGE of different SA variants.
Fig. S2. SMFS measurements with direct covalent attachment of the biotinylated ddFLN4 domain to the cantilever tip.
Fig. S3. Exemplary force extension traces.
Fig. S4. Dynamic force spectrum.
Fig. S5. Structure of biotin with the adjacent linker and illustration of the simulation box.
Fig. S6. SMD force histograms.
Fig. S7. Structure of the SA/biotin complex during L3/4 loop opening.
Fig. S8. Angle metric for L3/4 loop opening.
Fig. S9. Distance metric for L3/4 loop opening.
Table S1. Fit parameters for the Bell-Evans distributions shown in the main text.
Note S1. Fit parameters of Bell-Evans distributions.
Note S2. Sequences of protein constructs.

Other Supplementary Material for this manuscript includes the following:

(available at advances.sciencemag.org/cgi/content/full/6/13/eaay5999/DC1)

Movie S1 (.mov format). SA's crystal structure with highlighted amine groups.
Movie S2 (.mov format). Exemplary SMD: Holding biotin, pulling on the C terminus of SA subunit A.
Movie S3 (.mov format). Exemplary SMD: Holding biotin, pulling on the C terminus of SA subunit B.
Movie S4 (.mov format). Exemplary SMD: Holding biotin, pulling on the C terminus of SA subunit C.
Movie S5 (.mov format). Exemplary SMD: Holding biotin, pulling on the C-terminus of SA subunit D.

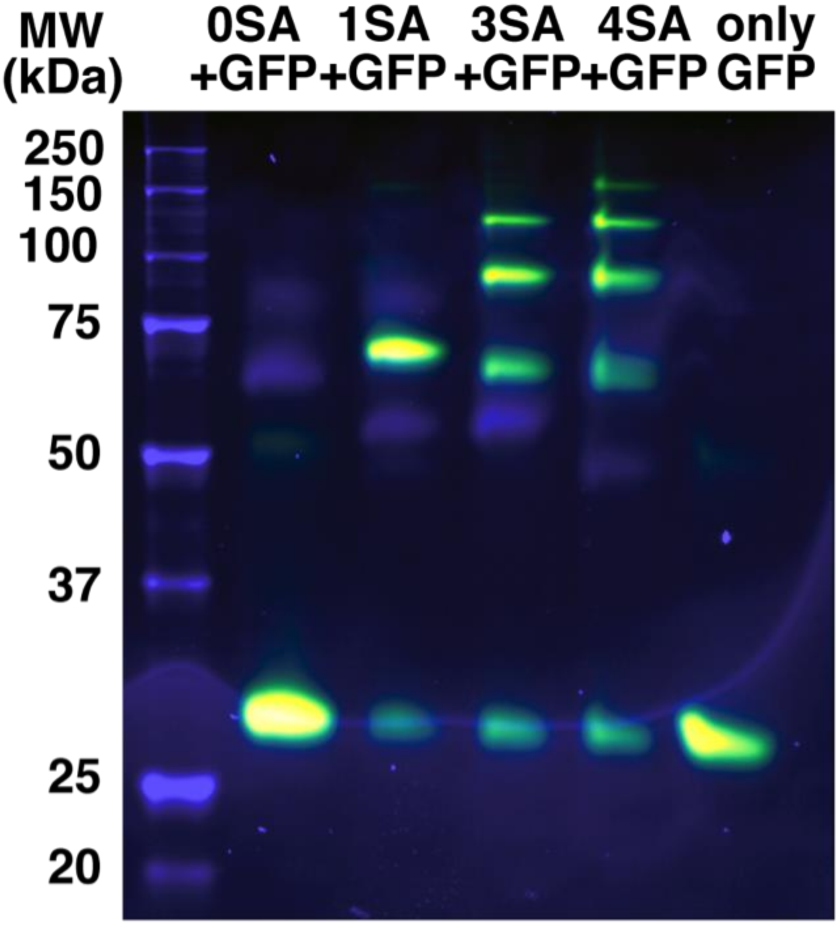

Fig. S1. SDS-PAGE of different SA variants. We mixed biotinylated GFP and SA such that the binding is not saturated. To preserve the structure of the proteins, the samples were loaded onto the gel without heating. The gel was imaged using illumination in the UV (blue) and at 488 nm (green). Obviously, 0SA is unable to bind biotinylated GFP, 1SA can bind only one, 3SA up to three and 4SA up to four biotinylated GFPs. The incomplete biotinylation results in GFP bands at about 27 kDa for all SA variants, even though the functional SA variants are not saturated. As the proteins were not heated prior to loading on the gel, the tetrameric structure is preserved and the SA bands appear at different positions on the gel. Futhermore, an additional band is visible at around 100 kDa, which we attribute to SA-octamers formed by disulfide bridges between the unique cysteines of two tetramers.

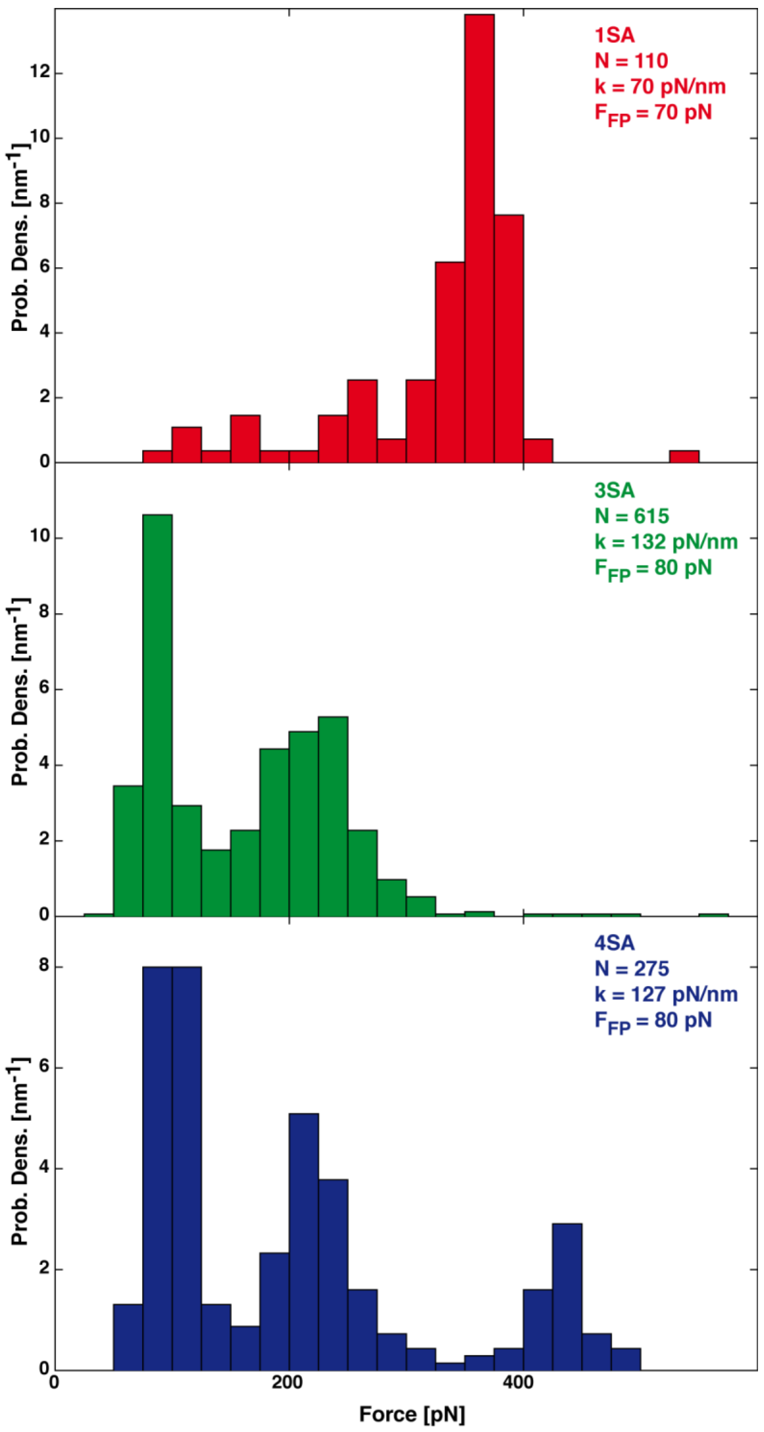

Fig. S2. SMFS measurements with direct covalent attachment of the biotinylated ddFLN4 domain to the cantilever tip. Data were recorded with different AFM cantilevers of spring constants *k*. Therefore, absolute values of the forces are subject to calibration errors, but can be put in relation by comparing the corresponding unfolding forces of the fingerprint domain F_{FP}. *E.g.*, for the 1SA measurement, the second step of the ddFLN4 unfolding was observed at 70 pN. The peak of the rupture force histogram occurs at 380 pN. Correcting the ddFLN4 peak to 80 pN, the corrected rupture force of 420 pN agrees well with the last peak of the 4SA histogram.

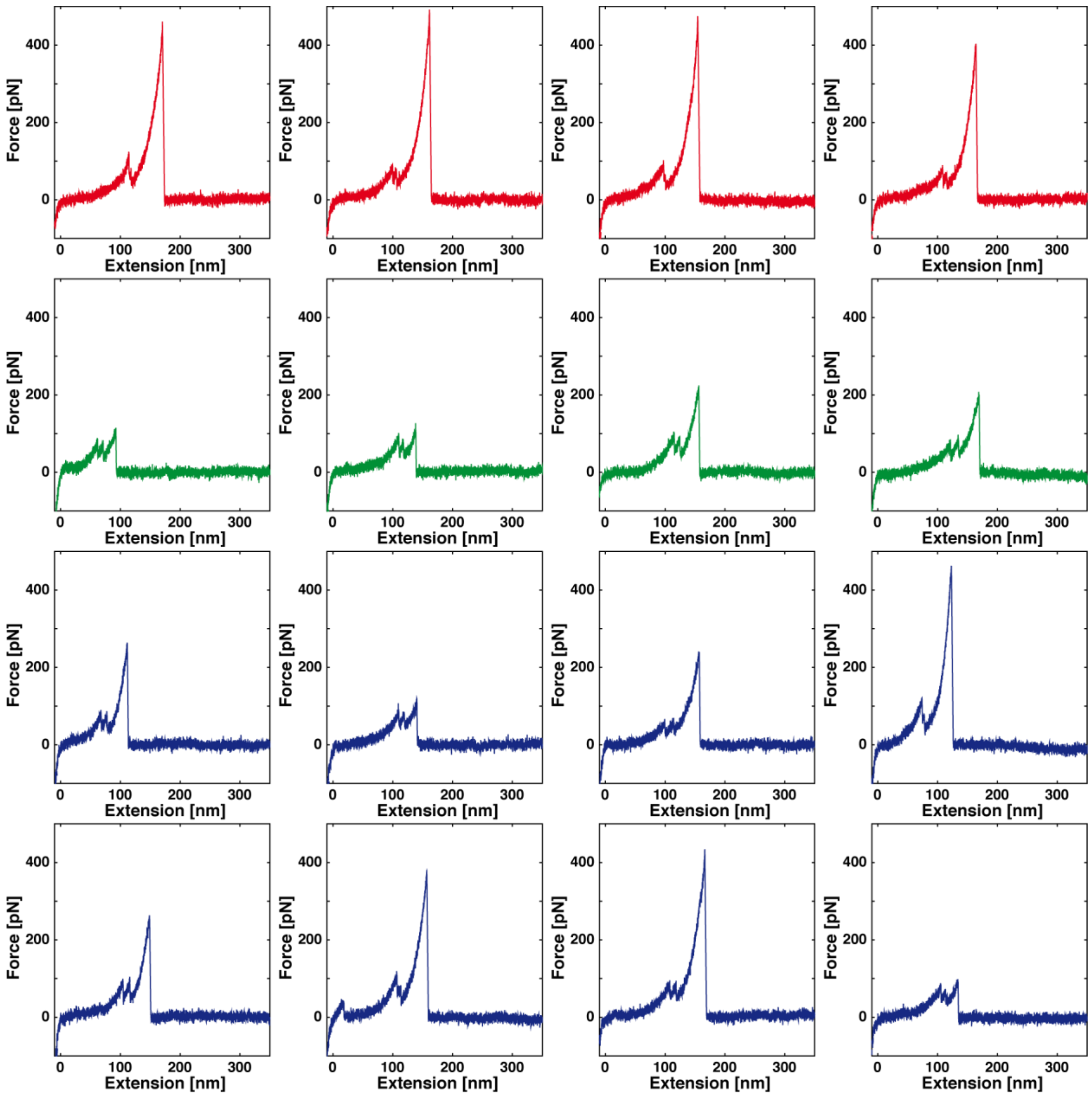

Fig. S3. Exemplary force extension traces. Force extension traces recorded on different surface areas are shown: Curves from the 1SA area in red, curves from the 3SA area in green, curves from the 4SA area in blue. All curves exhibit the distinct two-step unfolding pattern of the ddFLN4 domain.

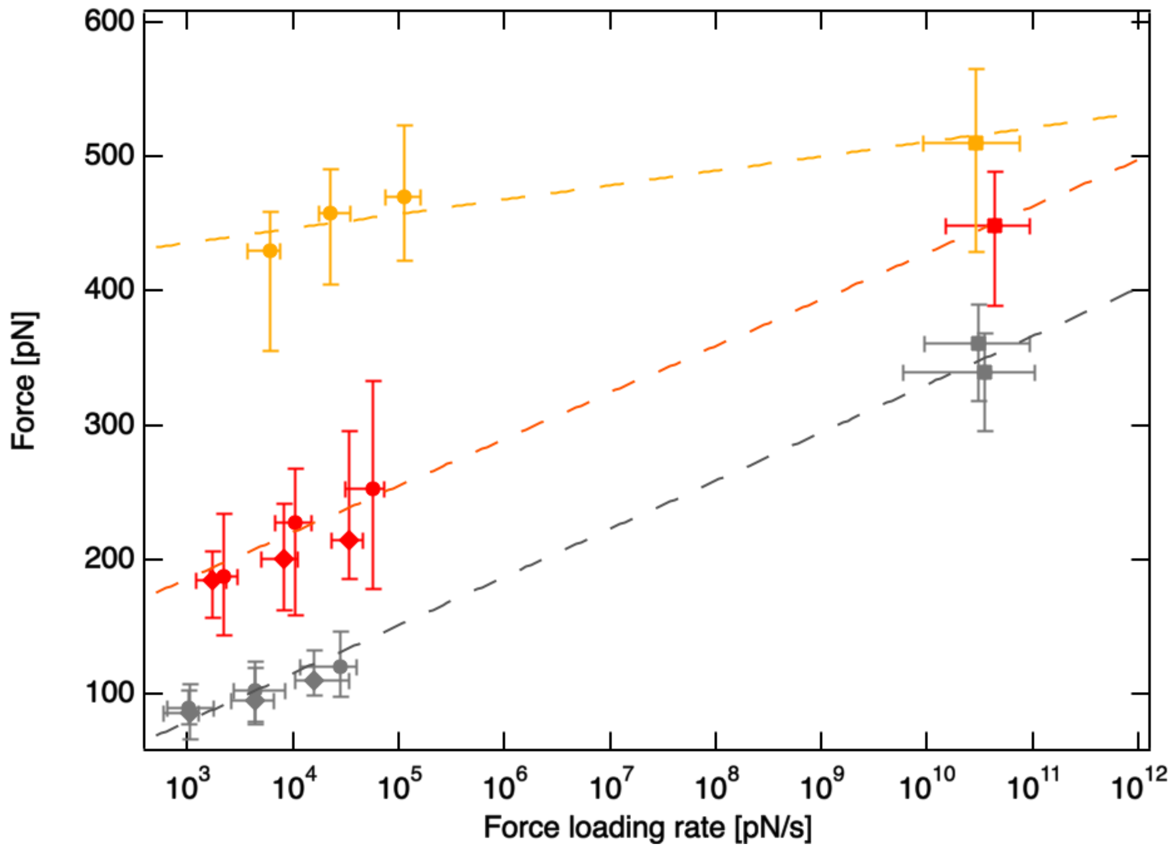

Fig. S4. Dynamic force spectrum. A dynamic force spectrum recorded for a surface with 4SA (circles) and a surface with 3SA (diamonds) is shown. Loading rates were varied applying retraction velocities of 200 nm/s, 800 nm/s and 3200 nm/s, respectively. For 4SA three peaks can be distinguished (yellow, red, grey) whereas for 3SA only the two peaks at lower forces occur (red, grey). The measurement of 3SA and 4SA was conducted on different surfaces with different AFM cantilever tips, which might account for the slight differences in loading rates and rupture forces. In addition, simulation data (squares) are plotted with the following coloring: Pulling of subunit D in yellow, pulling of subunit B in red, and pulling of subunit A and C in grey. Errors show the full width at half maximum of a kernel density estimation. Dashed lines show results for fitting the standard Bell-Evans model to the data.

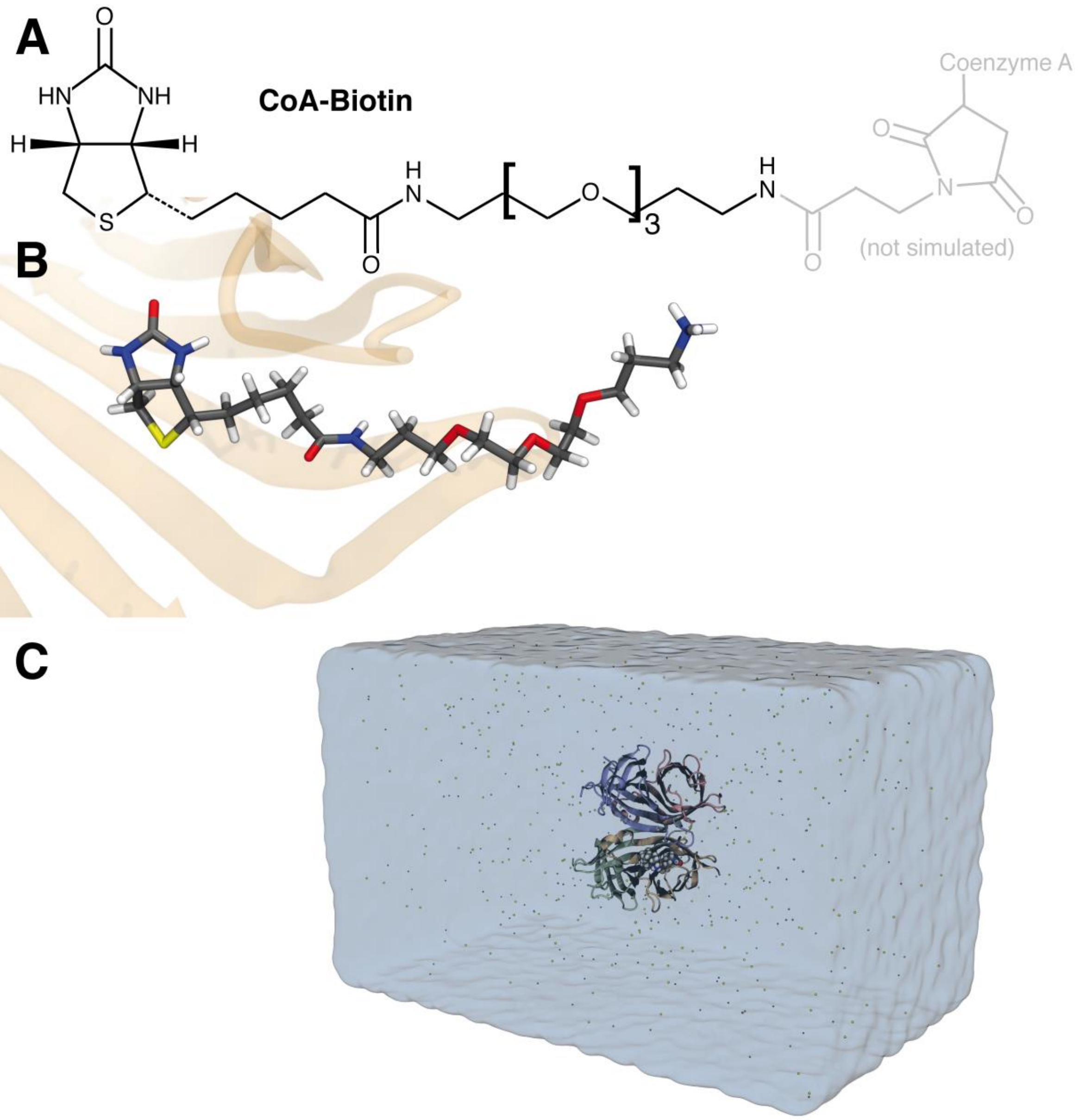

Fig. S5. Structure of biotin with the adjacent linker and illustration of the simulation box. (**A**) A Coenzyme A (CoA)-biotin (NEB, Ipswich, USA) was used for biotinylation of our protein constructs. The ureido ring and the tetrahydrothiophene ring of the biotin are on the left. The valeric acid, fused to the tetrahydrothiophene ring, which is present in pure biotin is reacted with an amine group and forms a peptide bond, connecting the biotin with a polyethylene glycol (PEG_3) linker. At the other end of the linker, a maleimide group is reacted to Coenzyme A. This latter group was however not considered in the SMD simulation (**B**). (**C**) Biotin (van der Waals representation) bound to SA (secondary structure representation with different colors assigned to different subunits) was solvated in a water box (transparent blue) containing a 0.15 mol/l sodium chloride (spheres) solution. The total simulation box was made by just over 275,000 atoms that were simulated explicitly.

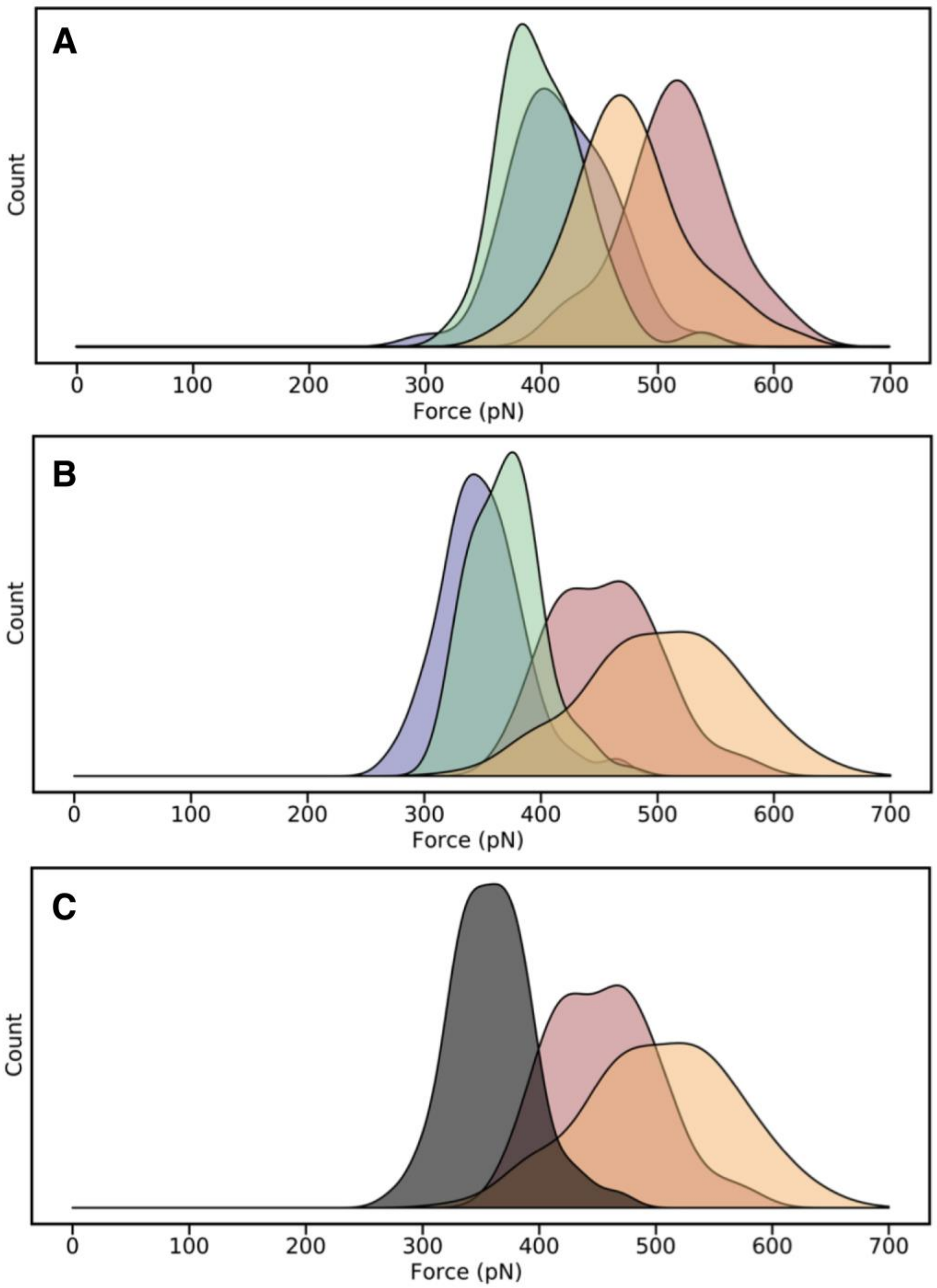

Fig. S6. SMD force histograms. Force histograms are shown for biotin unbinding for subunit D (yellow), subunit B (red), subunit C (green) and subunit A (blue) pulling. (**A**) For SMD simulations without the molecular linker adjacent to biotin, the force peaks are switched: subunit C is weaker than subunit A and subunit D is weaker than subunit B. (**B**) For comparison: combined histogram of the SMD simulations including the molecular linker. (**C**) Unbinding force histogram of SMD simulations with subunit A and subunit C combined: biotin unbinding for subunit D (yellow), subunit B (red) and combined plots for subunit C and subunit A (grey) pulling.

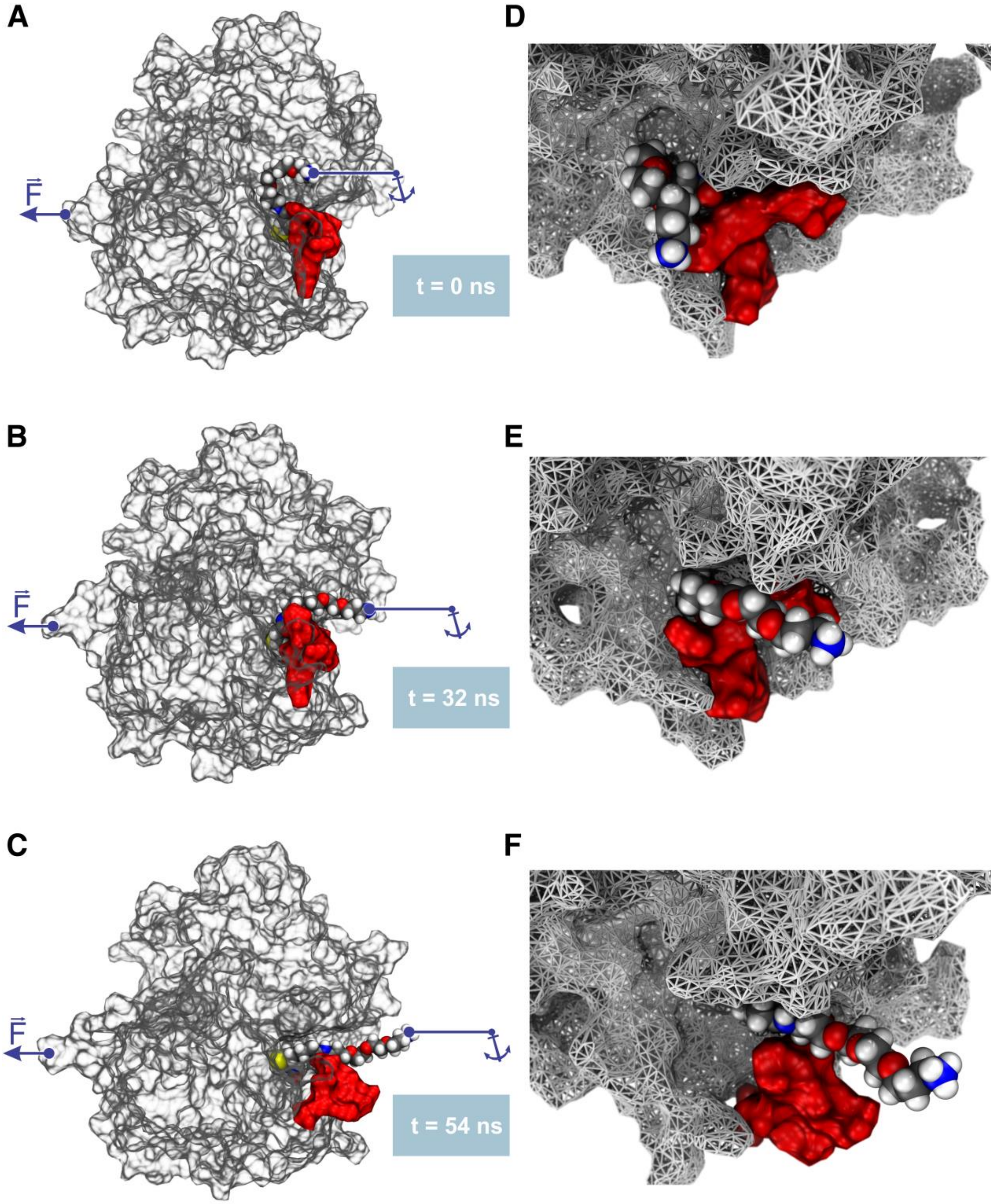

Fig. S7. Structure of the SA/biotin complex during L3/4 loop opening. The structure of SA (translucent grey) presents a flexible peptide loop between the third and the fourth β-strand (L3/4 loop) that closes over the binding pocket like a lid (red surface) and buries biotin inside the pocket. The structure of SA stretched *via* its subunit C and the end of the polymeric linker of biotin bound in subunit D is shown at initial configuration (**A**), prior to lid opening (**B**) and after lid opening just prior to bond rupture (**C**). At the same time frames, (**D**-**F**) shows a magnified and rotated view of the lid opening indicating how biotin and its linker molecule induce conformational changes in the binding pocket's lid.

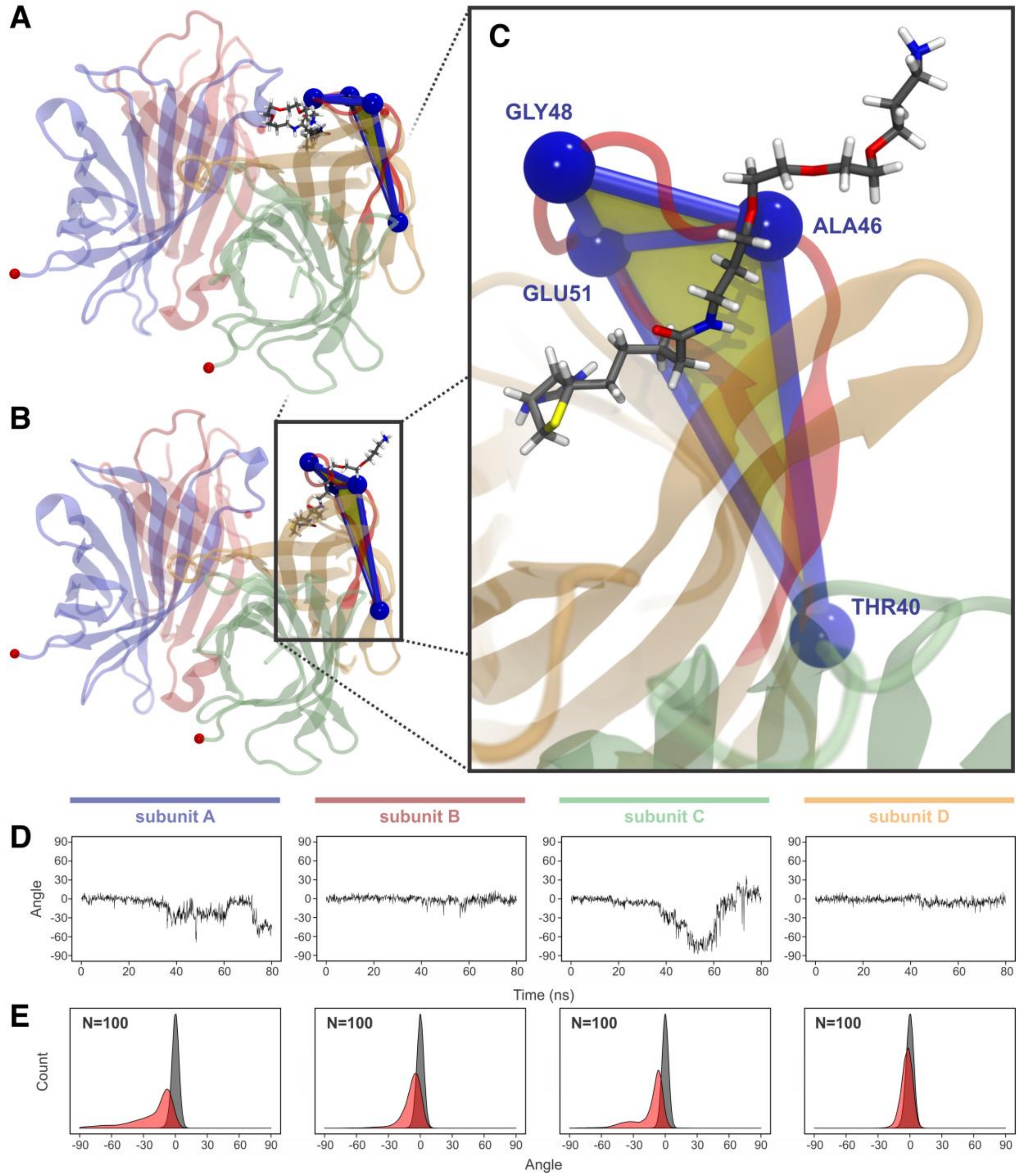

Fig. S8. Angle metric for L3/4 loop opening. Two planes (yellow) are introduced to provide a second metric for L3/4 loop opening. The first plane is being spanned through the α carbons of residues THR40, ALA46 and GLU51 (blue spheres) and the second through the α carbons of residues ALA46, GLY48 and GLU51. The dihedral angle between these two planes is used as indication for changes in the L3/4 loop conformation. The three parts depict the situations for the unloaded condition (**A**), under load prior to rupture (**B**) and just after biotin rupture (**C**). It indicates that upon unbinding the conformation of the L3/4 loop changes to an open conformation. (**D**) Exemplary plots of the dihedral angle measure for the L3/4 loop opening over time. (**E**) Histograms of the dihedral angle measured for the L3/4 loop opening for the first 10 ns of the simulation (unloaded condition, grey) and for 10 ns just prior to the point of rupture (loaded condition, red).

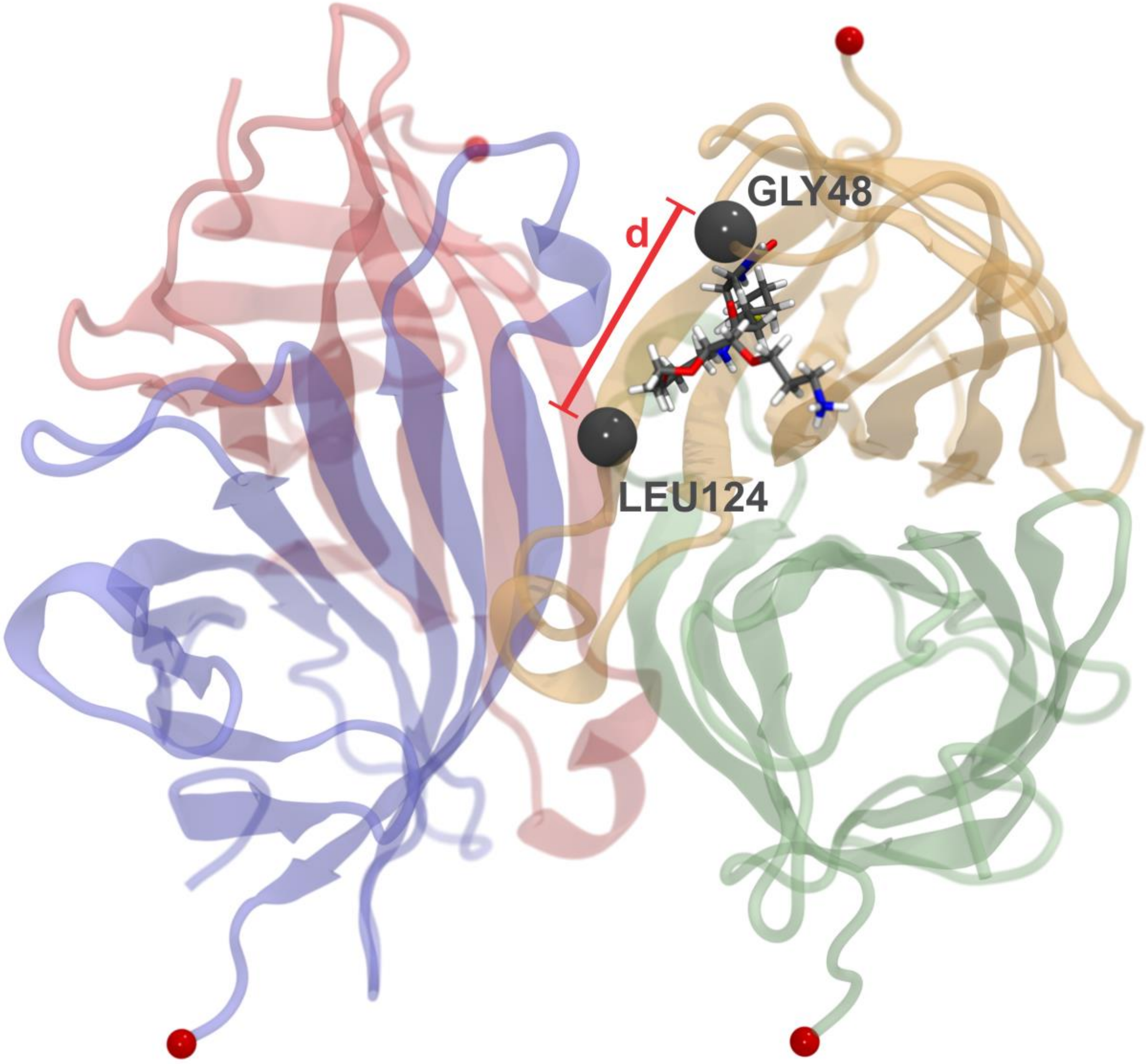

Fig. S9. Distance metric for L3/4 loop opening. The distance between the α carbon of residue GLY48 (black ball located in the center of the L3/4 loop) and the α carbon of residue LEU124 (black ball located in the middle of β-strand β8) on the opposite side of the binding pocket serves as one metric for L3/4 loop opening. These residues are tracked during each simulation for subunit D.

Table S1. Fit parameters for the Bell-Evans distributions shown in the main text. For the different peaks of the different SA variants, rupture force F_R, loading rate r, distance to transition state x_0, zero-force off-rate $k_{off,0}$ and weighting factor/relative occupancy are given. Note that the off-rate fitted for the 3rd peak is comparable to values for the natural off-rate found in the literature – most probably because the SA binding pocket is not deformed for this tethering geometry and stays in a state comparable to the unloaded conformation.

**SA**	**F_R [pN]**	**r [pN/s]**	**x_0 [nm]**	**$k_{off,0}$ [s^{-1}]**	**rel. occup.**
**1st peak**					
**4SA**	100	3800	0.27	0.28	37%
**3SA**	100	4390	0.27	0.31	39%
**2nd peak**					
**4SA**	210	7170	0.11	0.87	53%
**3SA**	210	7150	0.11	0.75	61%
**3rd peak**					
**4SA**	430	15100	0.17	1.2×10^{-5}	10%
**1SA**	440	16200	0.15	8.9×10^{-5}	100%

Supplementary Notes

Note S1. Fit parameters of Bell-Evans distributions

1SA data were fitted by a single Bell-Evans distribution

$$p_{a,b}(F) = a \cdot \exp(b \cdot x) \cdot \exp(a/b \cdot (1 - \exp(b \cdot x)))$$

with $a = k_{off,o}/r$ and $b = x_0/k_B T$, where $k_{off,0}$ is the zero-force off-rate, x_0 is the distance to the transition state, $k_B T$ the Boltzmann constant times temperature and r is the force loading rate.

3SA data were fitted by a double Bell-Evans distribution, *i.e.* by adding two Bell-Evans distributions weighted with two factors that add up to one

$$p_{a,b,c,d,e}(F) = e \cdot p_{a,b}(F) + (1 - e) \cdot p_{c,d}(F)$$

4SA data were fitted by a triple Bell-Evans distribution, *i.e.* by adding three Bell-Evans distributions weighted with three factors that add up to one

$$p_{a,b,c,d,e,f,g,h}(F) = e \cdot p_{a,b}(F) + f \cdot p_{c,d}(F) + (1 - e - f) \cdot p_{g,h}(F)$$

To determine the force-loading rate r is not straight-forward. For the single force-extension traces, the force-loading rate is determined from the slope of the force-extension trace 3 nm before the rupture peak. To obtain a mean value of the different r for the different force peaks, we first had to separate force-extension traces that belong to different force peaks. For this, we employed a kernel density estimate to fit to force histogram. The local minima between the different peaks were used as cut-off values: All force-extension traces with force values lower than the cut-off belong to one peak, all traces with forces higher than the cut-off value belong to the next peak. We then plotted a histogram of the force-loading rates corresponding to one peak and fitted it with a kernel density estimate. The maximum was then employed as loading rate r to convert the corresponding fitting parameter (a, c or g) into an off-rate $k_{off,0}$.

The fit parameters for the different SA variants agree well with each other for the different peaks of the histogram. Interestingly, only the zero-force off-rate for the highest force peak (3rd peak) are in the right order of magnitude compared to a conventional off-rate assay ($k_{off,exp}$=$6.1 \times 10^{-5} s^{-1}$). From this, we conclude that for the highest force peak, *i.e.* unbinding from subunit D, the mechanical integrity of the pocket is indeed not impeded too much by force application, so that the unbinding pathway is similar to the natural one.

Supplementary Notes

Note S2. Sequences of protein constructs

Functional SA subunit:
MEAGITGTWYNQLGSTFIVTAGADGALTGTYESAVGNAESRYVLTGRYDSAPATDGSG
TALGWTVAWKNNYRNAHSATTWSGQYVGGAEARINTQWLLTSGTTEANAWKSTLVG
HDTFTKVKPSAAS

Functional SA subunit with C-terminal cysteine (orange) and His-tag (green):
MEAGITGTWYNQLGSTFIVTAGADGALTGTYESAVGNAESRYVLTGRYDSAPATDGSG
TALGWTVAWKNNYRNAHSATTWSGQYVGGAEARINTQWLLTSGTTEANAWKSTLVG
HDTFTKVKPSAASCLEHHHHHH

Non-functional SA subunit (mutated residues in red):
MEAGITGTWYAQLGDTFIVTAGADGALTGTYEAAVGNAESRYVLTGRYDSAPATDGSG
TALGWTVAWKNNYRNAHSATTWSGQYVGGAEARINTQWLLTSGTTEANAWKSTLVG
HDTFTKVKPSAAS

Non-functional SA subunit with C-terminal cysteine (orange) and His-tag (green; mutated residues in red):
MEAGITGTWYAQLGDTFIVTAGADGALTGTYEAAVGNAESRYVLTGRYDSAPATDGSG
TALGWTVAWKNNYRNAHSATTWSGQYVGGAEARINTQWLLTSGTTEANAWKSTLVG
HDTFTKVKPSAASCLEHHHHHH

ddFLN4 construct with N-terminal Fgβ-motif (purple) and C-terminal His-tag (green) and ybbr-tag (blue; mutated cysteine in red):
MATNEEGFFSARGHRPLDGSGSGSGSAGTGSGADPEKSYAEGPGLDGGESFQPSKFKIH
AVDPDGVHRTDGGDGFVVTIEGPAPVDPVMVDNGDGTYDVEFEPKEAGDYVINLTLDG
DNVNGFPKTVTVKPAPSGHHHHHHGSDSLEFIASKLALPETGG

ddFLN4 construct with N-terminal ybbr-tag and His-tag (green) and C-terminal cysteine (orange; mutated cysteine in red):
MDSLEFIASKLAHHHHHHGSADPEKSYAEGPGLDGGESFQPSKFKIHAVDPDGVHRTDG
GDGFVVTIEGPAPVDPVMVDNGDGTYDVEFEPKEAGDYVINLTLDGDNVNGFPKTVTV
KPAPGSC

Green fluorescent protein (GFP) construct with N-terminal His-tag (green) and C-terminal ybbr-tag:
MGSSHHHHHHLEVLFQGPGHMCGSGSMSKGEELFTGVVPILVELDGDVNGHKFSVRGE
GEGDATIGKLTLKFISTTGKLPVPWPTLVTTLTYGVQCFSRYPDHMKRHDFFKSAMPEG
YVQERTISFKDDGKYKTRAVVKFEGDTLVNRIELKGTDFKEDGNILGHKLEYNFNSHNV
YITADKQKNGIKANFTVRHNVEDGSVQLADHYQQNTPIGDGPVLLPDNHYLSTQTVLSK
DPNEKRDHMVLHEYVNAAGITHGMDELYKSGSGSASDSLEFIASKLA
